# Studies on the Solubility of Terephthalic Acid in Ionic Liquids

**DOI:** 10.3390/molecules25010080

**Published:** 2019-12-24

**Authors:** Karolina Matuszek, Ewa Pankalla, Aleksander Grymel, Piotr Latos, Anna Chrobok

**Affiliations:** 1 Monash University, School of Chemistry, Clayton, VIC 3800, Australia; karolina.matuszek@monash.edu; 2 Grupa Azoty Zakłady Azotowe Kędzierzyn, S.A., Mostowa 30A, 47-220 Kędzierzyn-Koźle, Poland; ewa.pankalla@zak.com.pl (E.P.); ALEKSANDER.GRYMEL@GRUPAAZOTY.COM (A.G.); 3 Department of Chemical Organic Technology and Petrochemistry, Silesian University of Technology, Krzywoustego 4, 44-100 Gliwice, Poland; Piotr.Latos@polsl.pl

**Keywords:** terephthalic acid, ionic liquids, solubility

## Abstract

Low solubility of terephthalic acid in common solvents makes its industrial production very difficult and not environmentally benign. Ionic liquids are known for their extraordinary solvent properties, with capability to dissolve a wide variety of materials, from common solvents to cellulose, opening new possibilities to find more suitable solvents for terephthalic acid. This work presents studies on the solubility of terephthalic acid in ionic liquids, and demonstrates that terephthalic acid is soluble in ionic liquids, such as 1-ethyl-3-methylimidazolium diethylphosphate, 1-butyl-3-methylimidazolium acetate, and dialkylimidazolium chlorides up to four times higher than in DMSO. Additionally, the temperature effect and correlation of ionic liquid structure with solubility efficiency are discussed.

## 1. Introduction

Purified terephthalic acid (PTA) is a white, crystalline solid with negligible vapor pressure under standard conditions [1]. Nearly all PTA is consumed in polyester production, including polyester fiber and film, and polyethylene terephthalate (PET) resin. Nowadays, PTA is produced by the catalytic liquid-phase oxidation of p-xylene in acetic acid, in the presence of air, using a manganese or cobalt acetate catalyst. In 2011, global PTA capacity reached 28.8 million tons, with China being the main world producer [2].

Terephthalic acid is poorly soluble in organic solvents (Table 1) [1,3]. Among all tested solvents the best solubility of PTA was observed in DMSO (20 g of PTA per 100 g DMSO at 25 °C). PTA is also soluble in N-methyl-2-pyrolidone and dimethylamine, but the solubility at 90 °C is two times lower than in DMSO [3]. In general, the solubility of PTA in organic solvents is low and slowly increases with increasing temperature.

Low solubility of PTA in conventional solvents creates problems in industry during the purification and transformation of PTA into useful chemicals. The crude terephthalic acid (CTA) contains impurities like p-toluic acid and 4-carboxybenzaldehyde (CBA). CTA is purified by crystallization from water, dissolving CTA at 300 °C and elevated pressure; however, under these conditions the solubility is only 40 mass%. As a result, this methodology is expensive and energy consuming. This problem also arises in the other applications like chemical transformations, where low miscibility of PTA with other reagents results in low product rate. One of the examples is the production of esters of terephthalic acid as alternative plasticizers. Poor solubility of PTA in alcohols forces the use of higher pressures and temperatures in the esterification processes.

Therefore, alternative solvents, which can overcome dissolution limitations, are of high importance for the processing of PTA. Ionic liquids (ILs) with their unique properties can help to solve some of the described problems above [4,5,6,7,8]. Ionic liquids make promising alternatives to conventional solvents, and lead to a both greener and economically viable process. Ionic liquids as sustainable solvents play an important role in pharmacological development [9,10] and in the chemical industry [11]. Various approaches of implementing ionic liquids in organic synthesis as solvents and catalysts were also demonstrated [12,13].

**Figure 1:**
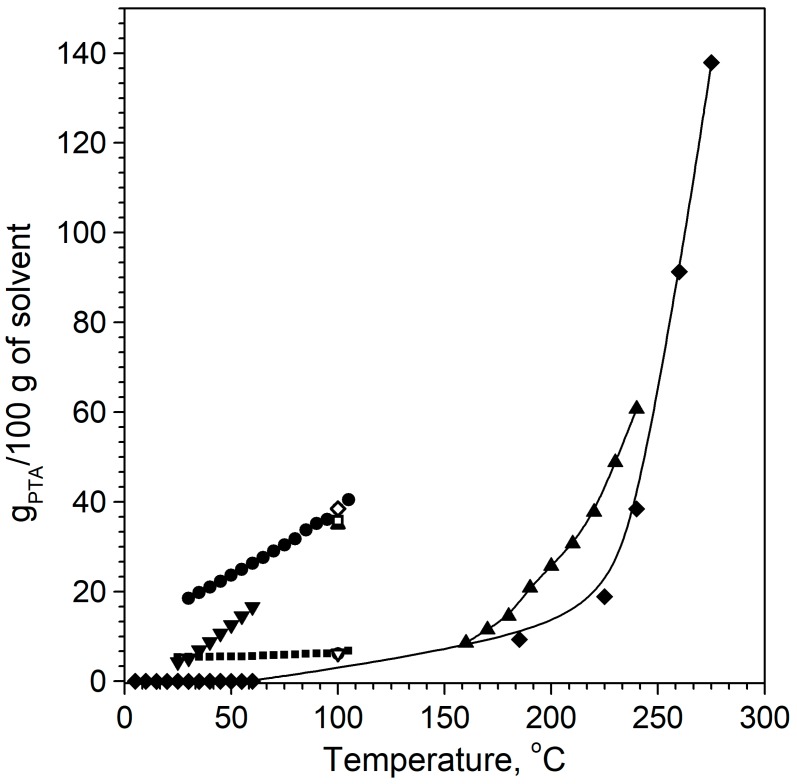
Solubility of PTA in ionic liquids and conventional solvents; figure adapted from Rogers, R. D et al. US 2010174111 A1, 20109 (⋄) [C_2_mim][Et2PO4], (Δ) [C_4_mim]Cl, (□) [C_2_mim]Cl, (∇) [C_2_mpy][EtSO_4_], (∘) [C_2_mim][EtSO_4_], (◆) water, (▴) acetic acid, (▪) DMF, (▾)N-Methyl-2-pyrrolidone, (•) DMSO.

Literature concerning solubilities of PTA in ionic liquids is scarce. However, several patents concerning purification of the CTA from by-products using ionic liquids exists, and solubility data are presented [14,15,16]. Among them is a patent concerning the purification of aryl carboxylic acids published in 2010, which reports that the impure acid is dissolved or dispersed in an ionic liquid, which is followed by the addition of a non-solvent to precipitate the acid while other impurities remain dissolved. In this case, the term “non-solvent” defines a compound that is highly soluble in ionic liquids with little or no solubility in aryl carboxylic acid [14]. A detailed graph comparing the solubility of PTA in ionic liquids and conventional solvents was provided (Figure 1) [5]. High solubility of PTA in several ionic liquids was shown in relatively low temperatures. According to this data, PTA can be dissolved in ionic liquids to a greater extent than in solvents such as DMSO, DMF, or water. Among all studied ionic liquids, the solubility of PTA was shown in the following order: 1-ethyl-3-methylimidazolium diethylphosphate [C_2_mim][Et_2_PO_4_] > 1-butyl-3-methylimidazolium chloride [C_4_mim]Cl > 1-ethyl-3-methylimidazolium chloride [C_2_mim]Cl > *N*-ethyl-2-methylpyridinium ethyl sulfate [C_2_mpy][EtSO_4_] > 1-ethyl-3-methylimidazolium ethyl sulfate [C_2_mim][EtSO_4_]. For example, 37 mass% of PTA was dissolved in [C_2_mim][Et_2_PO_4_] at 100 °C, while in DMSO only 15 mass% could be dissolved.

The next patent, published in 2013, describes purification of CTA containing CBA as impurity with ionic liquids [1]. After 2 h, the mixture of CTA and an ionic liquid was cooled down to room temperature and the solid (PTA) was filtered off. The best results were obtained with the application of trihexyl(tetradecyl)phosphine bromide (Cyphos 102), trihexyl(tetradecyl)phosphine chloride (Cyphos 101), 1-butyl-1-methylpyrrolidinium bis(trifluoromethanesulfonyl)imide ([bmpyr][NTf_2_]) and 1-butyl-3-methylimidazolium acetate ([bmim][OAc]). Application of these ionic liquids led to the removal of 99%, 97%, 93%, and 90% of CBA, respectively. Information about the exact amounts of PTA dissolved in ionic liquids was not presented. 

In 2015, a patent was issued that involved the separation of aryl carboxylic acids from a mixture comprising at least two acids [16]. The separation process is based on heating the mixture of at least two aryl carboxylic acids with an ionic liquid, and cooling down using fractional crystallization to precipitate the desired acid. Information about solubility of PTA in ionic liquids can be found in several examples in the patent: A total of 59.28 g of PTA/100 g of choline chloride at 224 °C, 47.56 g of PTA/100g of 1-butyl-3-methylimidazolium chloride at 160 °C, 6.25 g of PTA/100g of trihexyltetradecylphosphonium bromide at 160 °C, 45.10 g of PTA/100 g of choline bromide at 220 °C, 37.89 g of PTA/100 g of 1-butyl-3-methylimidazolium bromide at 180 °C, 17.54 g of PTA/100 g of 1-butyl-3-methylimidazolium methanesulfonate at 120 °C.

In summary, patents, which describe purification of CTA from typical impurities, such as CBA or p-toluic acid, provide some data concerning the solubility of PTA in ionic liquids. This data shows that the solubility of PTA in ionic liquids is higher than in conventional solvents; however, in many cases there is a lack of detailed information concerning the amounts of PTA dissolved in ionic liquids at a given temperature.

This work provides detailed research concerning the solubility of terephthalic acid in ionic liquids as a function of temperature, and also discusses the influence of the cation and anion structure on the solubility of PTA in various ionic liquids.

## 2. Results and Discussion

Ionic liquids used in this work were chosen based on the patent literature described above [9,10,11]. Solubility studies were conducted at temperatures between 25 and 100 °C and the obtained results are summarized in Table 2 in order of decreasing solubility.

Among the studied ionic liquids, the best results were obtained for [C_2_mim][Et_2_PO_4_]. At room temperature, 52.2 g_PTA_/100 g_IL_ was dissolved, and 63.4 g_PTA_/100 g_IL_ at 100 °C, which is two times greater than in DMSO (29.4 g_PTA_/100 g_DMSO_ at 100 °C). High solubilities were also observed for [C_4_mim][OAc] (57.1 g_PTA_/100 g_IL_, 100 °C) and the dialkylimidazolium chlorides [C_2_mim]Cl (42.9 g_PTA_/100 g_IL_, 100 °C), [C_4_mim]Cl (34.0 g_PTA_/100 g_IL_, 100 °C) and [C_6_mim]Cl (34.2 g_PTA_/100 g_IL_, 100 °C). All other tested ionic liquids exhibited lower solubility than DMSO. It can be concluded that the highest solubilities of PTA were found in ionic liquids that were aprotic, contained Lewis base properties (similar to DMSO [17]), and possessed chloride, acetate, or diethylphosphate anions. Protic ionic liquids with Lewis base properties ([Hmim][OAc]) also show relatively high PTA solubility (24.7 g_PTA_/100 g_IL_, 100 °C); although, this is lower than its aprotic homologue ([C_4_mim][OAc]), most probably due to the weak Brønsted acid properties of the free proton on the nitrogen atom in the [Hmim]^+^cation.

The solubility of terephthalic acid in ionic liquids slowly increases with temperature (52.2 g_PTA_/100 g_IL_ at 25 °C, and 63.4 g_PTA_/100 g_IL_ at 100 °C). This effect is much more pronounced in the case of conventional solvents (0.1 g_PTA_/100 g_MeOH_ at 25 °C, and 3.1 g_PTA_/100 g_MeOH_ at 150 °C). This can be explained by the solubility in conventional solvents at room temperature being relatively low, with only the increase of the average kinetic energy, caused by increase of temperature, allowing the solvent molecules to overcome intermolecular attraction and break apart the terephthalic acid molecules. On the other hand, some ionic liquids can already overcome those attraction forces at room temperature and; therefore, temperature only slightly increases the solubility. 

### 2.1. The Influence of the Structure of Cation in Ionic Liquids

**Figure 2:**
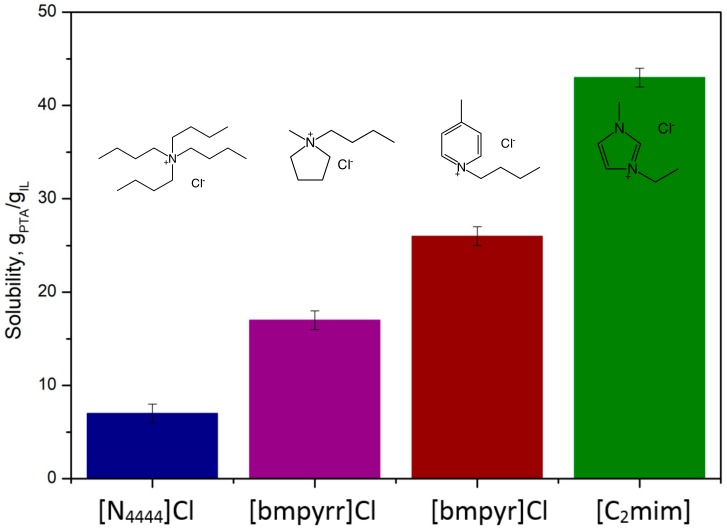
The influence of the structure of cation in ionic liquids on the solubility of PTA at 100 °C.

It was found that the structure of cation in the ionic liquid affects the PTA solubility. Four ionic liquids with the same chloride anion and with different structures of the cation were compared in Figure 2. Tested cations represent a variety of structures, such as aromatic—1-butyl-4-methylpiridinium and 1-ethyl-3-methylimidazolium, alicyclic—1-butyl-1-methylpyrrolidinium and aliphatic—tetrabutylammonium. Ionic liquids with aliphatic substituents in the ammonium cation exhibit the lowest solubility of PTA, only 6.7 g_PTA_/100 g_IL_ at 100 °C. The use of a cyclic, non-aromatic cation allows for slightly better results: 17.4 g_PTA_/100 g_IL_ at 100 °C. Ionic liquids with aromatic cations turned out to be the most effective (42.9 g_PTA_/100 g_IL_). Most likely, this is due to the effect of π–π interactions between aromatic rings located in both ionic liquid cation and PTA [18].

### 2.2. The Influence of the Alkyl Side Chain Length in the Cation

**Figure 3:**
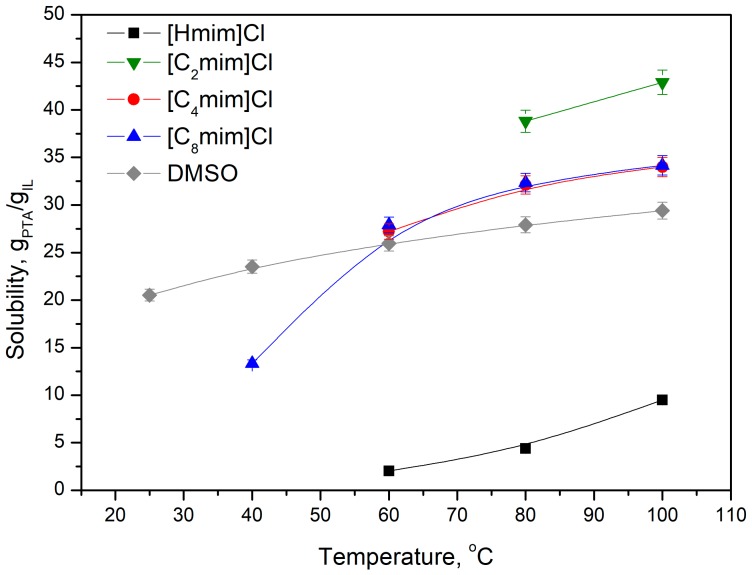
The influence of the alkyl chain length in the 1-alkyl-3-methylimidazolium cation on the solubility of PTA.

The dialkylimidazolium cation possesses two alkyl substituents in the structure, which influence the physicochemical properties of ionic liquid (e.g., density, melting point, and viscosity). Ionic liquids based on the 1-alkyl-3-methylimidazium cation with various side alkyl chain length—ethyl [C_2_mim]^+^, butyl [C_4_mim]^+^, hexyl [C_6_mim]^+^, octyl [C_8_mim]^+^—and also an ionic liquid with a proton instead of an alkyl chain [Hmim]^+^, were selected for these studies. Obtained results are presented in Figure 3. The first three homologs are crystalline solids at room temperature. The elongation of the alkyl chain to six carbons causes a symmetry disorder and, consequently, [C_6_mim]Cl and [C_8_mim]Cl are very viscous liquids, with a viscosity of 715 and 337 cP at 25 °C, respectively. The PTA solubility decreases as the length of the alkyl chain increases from ethyl to butyl or octyl. Similar observations were noted for ILs and water [19]. Better solubility of PTA in the 1-ethyl-3-methylimidazolium chloride can be attributed to the higher charge density and polarity, compared to the longer homologues. 

### 2.3. The Influence of the Structure of Anion in Ionic Liquids

**Figure 4:**
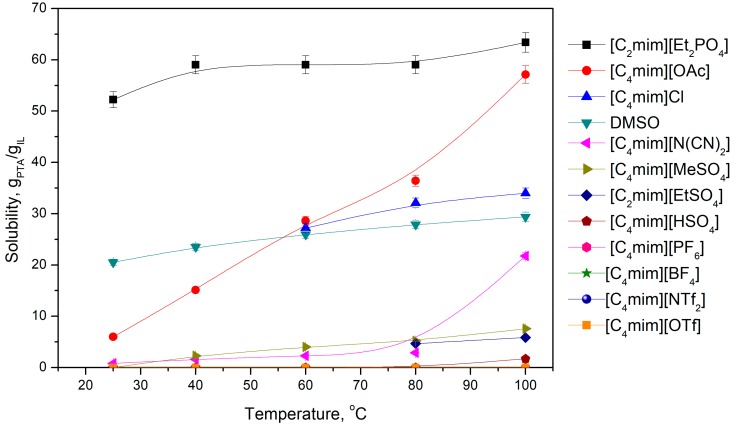
The influence of anion structure on the solubility of PTA.

To observe the influence of the anion on the solubility of PTA, a wide range of ionic liquids with the same 1-methyl-3-butylimidazolium cation and different anions were studied. The anions showing neutral (in the acid/base sense: [BF_4_]^−^, [PF_6_]^−^, [CH_3_SO_3_]^−^, [NTf_2_]^−^), acidic ([OTf]^−^, [OAc(HOAc)_2_]^−^, [(HSO_4_)(H_2_SO_4_)_2_]^−^), amphoteric ([HSO_4_]^−^), as well as basic ([CH_3_COO]^−^, Cl^−^, [Et_2_PO_4_]^−^, [N(CN)_2_]^−^) properties were selected [17]. The results of PTA solubility as a function of temperature are presented in Figure 4. 

According to the data presented in this and previous paragraphs, anion structure is crucial in deciding the solubility of PTA. This can be expected, since the chemical properties of ionic liquids are mainly determined by the structure of the anion [20]. The best results were achieved using ionic liquids with diethylphosphate (V) and acetate anions, followed by chloride. Ionic liquids based on these anions possess proton acceptor abilities, which might play a role in the interaction with carboxylic groups from PTA; therefore, enhancing its solubility. 

Ionic liquid with the [N(CN)_2_]^−^ anion, which exhibit Lewis base properties, has a moderate ability to dissolve PTA. Nevertheless, the solubility is still better than that for ionic liquids showing weak acidic [EtSO_3_]^−^ or amphoteric [HSO_4_]^−^ properties.

Ionic liquids constructed with acid/base neutral anions—[BF_4_]^−^, [PF_6_]^−^, [CH_3_SO_3_]^−^, [NTf_2_]^−^—do not dissolve PTA at all, irrespective of temperature.

### 2.4. The Solubility of Terephthalic Acid in Protic and Aprotic Ionic Liquids

**Figure 5:**
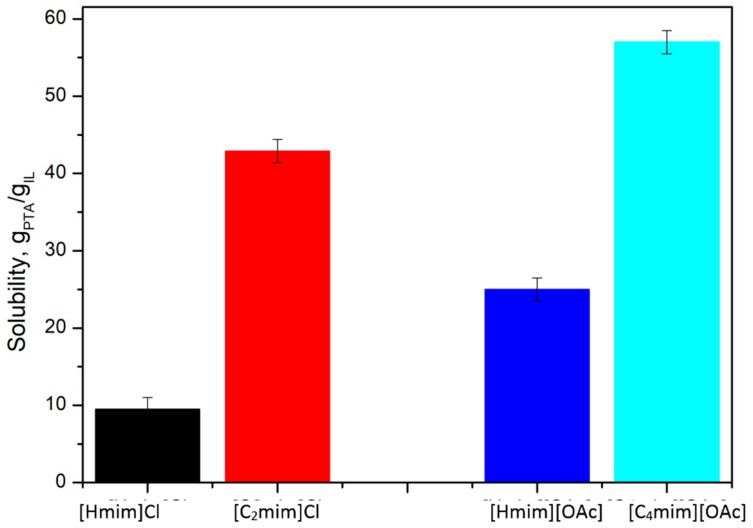
The comparison of the solubility of PTA in protic and aprotic ionic liquids.

Aprotic ionic liquids, based on acetate and chloride anions showing good PTA solubility, encouraged us to test their protic analogues, which are cheaper and easier to manufacture. These analogues are formed in a simple reaction between acid and base, for example, acetic acid and 1-methylimidazole. For this purpose, [Hmim][OAc] and [Hmim]Cl ionic liquids were synthesized. Unfortunately, protic ionic liquids show a lower solubility of PTA than their aprotic analogues (Figure 5). It is presumed to be caused by their higher Brønsted acidity, which arises from the presence of a labile proton on the nitrogen atom in the cation.

## 3. Materials and Methods

Ionic liquids used in this study: 1-ethyl-3-methylimidazolium diethylphosphate [C_2_mim][Et_2_PO_4_], 1-butyl-3-methylimidazolium acetate [C_4_mim][OAc], 1-ethyl-3-methylimidazolium chloride [C_2_mim]Cl, 1-butyl-3-methylimidazolium dicyanamide [C_4_mim][N(CN)_2_], 1-butyl-1-methylpyrrolidinium chloride [bmpyr]Cl, tetrabutylammonium bromide [N_4444_]Br, tetradecyl(trihexyl)phosphonium chloride [P_14666_]Cl, tetrabutylammonium chloride [N_4444_]Cl, 1-butyl-3-methylimidazolium methyl sulfate [C_4_mim][MeSO_4_], 1-ethyl-3-methylimidazolium ethyl sulfate [C_2_mim][EtSO_4_], tetradecyl(trihexyl)phosphonium bromide [P_14666_]Br, 1-butyl-3-methylimidazolium hydrogen sulfate [C_4_mim][HSO_4_], 1-butyl-3-methylimidazolium trifluoromethanesulfonate [C_4_mim][OTf], and 1-butyl-3-methylimidazolium hexafluorophosphate [C_4_mim][PF_6_] were purchased from Sigma-Aldrich and dried before use on the Schlenk line (40 °C, 0.5 mbar, 12 h). Other ionic liquids: 1-butyl-3-methylimidazolium chloride [C_4_mim]Cl, 1-hexyl-3-methylimidazolium chloride [C_6_mim]Cl, 1-octyl-3-methylimidazolium chloride [C_8_mim]Cl, 1-methylimidazolium acetate [Hmim][OAc], 1-methylimidazinium chloride [Hmim]Cl, 1-butyl-3-methylimidazolium bis(trifluoromethylsulfonyl)imide [C_4_mim][NTf_2_], 1-butyl-3-methylimidazolium tetrafluoroborate [C_4_mim][BF_4_], and 1-methylimidazolium hydrogen sulfate [Hmim][HSO_4_] were synthetized according to the known procedures [7]. PTA was purchased from Sigma-Aldrich and used without further purification. 

Solubility measurements: A total of 1 g of ionic liquid was placed in the round bottom flask and then PTA was added in small portions (0.01 g). The mixture was closed under argon atmosphere and stirred using a thermostatic magnetic stirrer for 1 h. When the mixture became homogeneous, the next portion of PTA (0.01 g) was added; if not, the temperature was raised. Measurements were carried out sequentially at 25, 40, 60, 80, and 100 °C.

## 4. Conclusions

This study systematically expands on the limited existing knowledge concerning the solubility of PTA in ionic liquids. It was confirmed that ionic liquids exhibit a high capacity to dissolve PTA, almost twice higher than the best conventional solvent—DMSO. Ionic liquids based on diethylphosphate, acetate, and chloride anions with Lewis base properties were the most effective. Solubility in ionic liquids decreases in the following order: [C_2_mim][Et_2_PO_4_] > [C_4_mim][OAc] > [C_2_mim]Cl > [C_4_mim]Cl > [C_6_mim]Cl. Additionally, it was observed that ionic liquids containing an aromatic cation, preferably dialkylimidazolium, performed better than other studied cations. The longer the alkyl substituent in the 1-alkyl-3-methylimidazolium cation, the lower the charge density and polarity, which impedes the solubility of PTA. Additionally, solubility of PTA in ionic liquids strongly depends on the anion structure; the most effective ones possessing an anion with Lewis base properties. In summary, ionic liquids have a high potential to dissolve PTA and can; therefore, be an effective alternative for conventional solvents.

## Figures and Tables

**Table 1:** Solubility of PTA (terephthalic acid) in organic solvents (g_PTA_/100 g solvent) [1].

Solvent	Temperature		
	25 °C	150 °C	200 °C
Water	0.0017	0.24	1.7
Methanol	0.1	3.1	lack of data
DMF	7.4	lack of data	lack of data
DMSO	20.0	lack of data	lack of data

DMF, dimethylformamide; DMSO, dimethyl sulfoxide.

**Table 2:** Solubility of PTA in ionic liquids.

No.	Ionic Liquid	PTA Solubility g/100 g IL (±0.5 g)				
		25 °C	40 °C	60 °C	80 °C	100 °C
1	1-ethyl-3-methylimidazolium diethylphosphate [C_2_mim][Et_2_PO_4_]	52.2	59.0	60.3	62.1	63.4
2	1-butyl-3-methylimidazolium acetate [C_4_mim][OAc]	6.0	15.1	28.6	36.4	57.1
3	1-ethyl-3-methylimidazolium chloride [C_2_mim]Cl	solid	solid	solid	38.8	42.9
4	1-butyl-3-methylimidazolium chloride [C_4_mim]Cl	solid	solid	27.2	32.1	34.0
5	1-hexyl-3-methylimidazolium chloride [C_6_mim]Cl	h.v.	13.3	27.9	32.4	34.2
6	1-octyl-3-methylimidazolium chloride [C_8_mim]Cl	h.v.	h.v.	5.6	15.6	30.9
7	DMSO	20.5	23.5	25.6	27.9	29.4
8	1-butyl-3-methylimidazolium dicyanamide [C_4_mim][N(CN)_2_]	0.8	1.6	2.3	2.9	21.8
9	1-butyl-1-methylpyridinum chloride [bmpyr]Cl	solid	solid	solid	solid	25.9
10	1-methylimidazolium acetate [Hmim][OAc]	0	3.0	6.8	10.7	24.7
11	1-butyl-1-methylpyrrolidinium chloride [bmpyrr]Cl	solid	solid	solid	solid	17.4
12	Tetrabutylammonium bromide[N_4,4,4,4_]Br	solid	solid	solid	solid	19.1
13	Tetradecyl(trihexyl)phosphonium chloride [P_14,6,6,6_]Cl	0	4.4	6.3	7.9	13.2
14	Tetrabutylammonium chloride [N_4,4,4,4_]Cl	solid	solid	solid	solid	6.7
15	1-methylimidazolium chloride [Hmim]Cl	solid	solid	2.0	4.4	9.5
16	1-butyl-3-methylimidazolium methylsulfate [C_4_mim][MeSO_4_]	0	2.3	4.0	5.2	7.6
17	1-ethyl-3-methylimidazolium ethylsulfate [C_2_mim][EtSO_4_]	0	0	0	4.7	5.9
18	Tetradecyl(trihexyl)phosphonium bromide [P_14,6,6,6_]Br	0	0	1.4	2.8	5.2
19	1-butyl-3-methylimidazolium hydrogensulfate [C_4_mim][HSO_4_]	h.v.	0	0	0	1.7
20	1-butyl-3-methylimidazolium bis(trifluoromethylsulfonyl)imide [C_4_mim][NTf_2_]	0	0	0	0	0
21	1-butyl-3-methylimidazolium trifluoromethanesulfonate [C_4_mim][OTf]	0	0	0	0	0
22	1-butyl-3-methylimidazolium hexafluorophosphate [C_4_mim][PF_6_]	0	0	0	0	0
23	1-butyl-3-methylimidazolium tetrafluoroborate [C_4_mim][BF_4_]	0	0	0	0	0
24	1-methylimidazolium hydrogen sulfate [Hmim][HSO_4_]	0	0	0	0	0

h.v., high viscosity at a given temperature.
